# Supplementary material for: Loss of Arginase 2 Promotes Lung Metastasis in immune-competent hosts via Nitric Oxide Synthase 2-Dependent Th17 Response
Source: bioRxiv. 2026 Jun 8:2026.06.04.730112. Preprint. [Version 1] doi: 10.64898/2026.06.04.730112 (PMC13278164; doi:10.64898/2026.06.04.730112)
Supplement: Supplementary file 1 [file media-1.pdf]

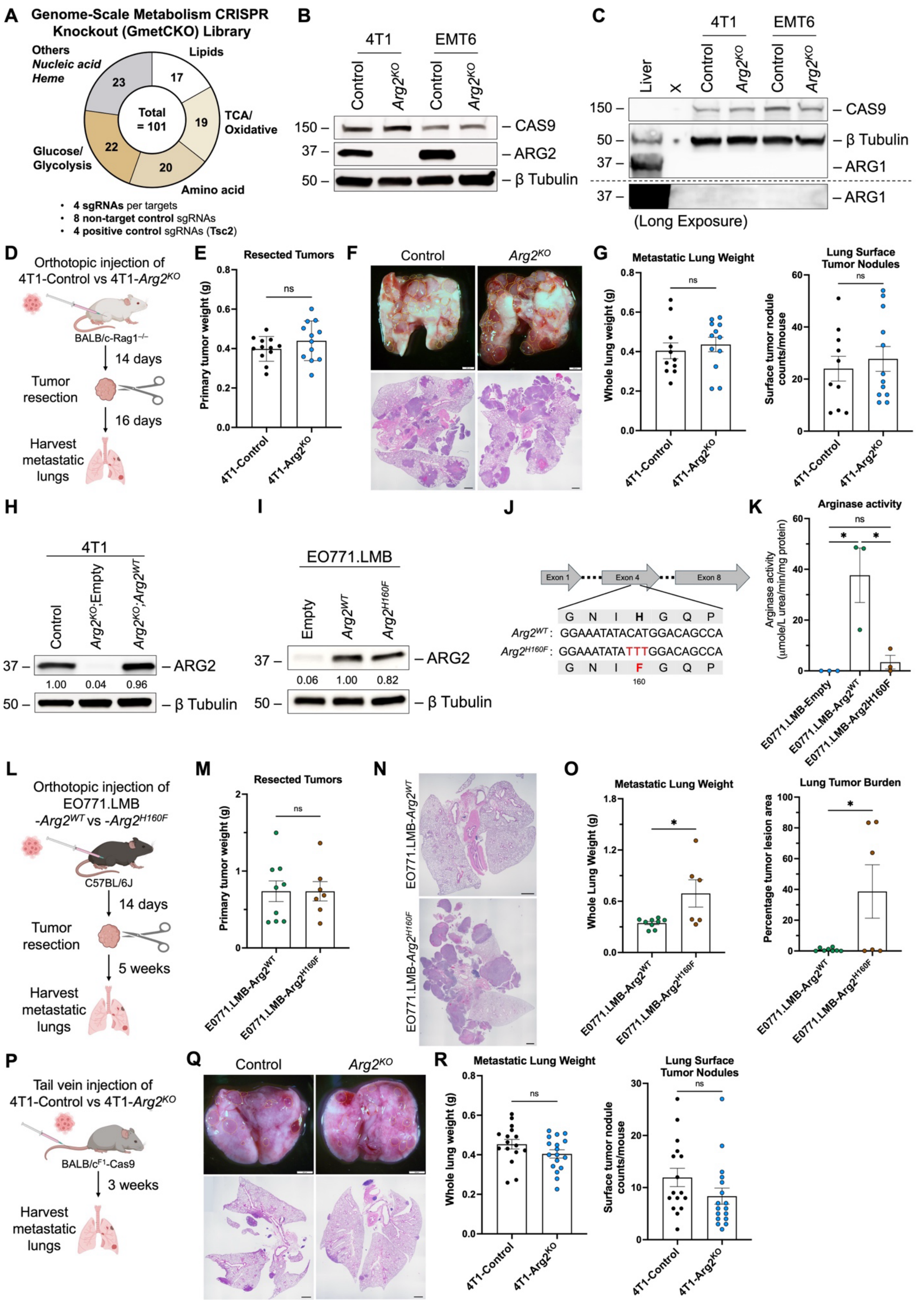

A Genome-Scale Metabolism CRISPR Knockout (GmetCKO) Library
Others Nucleic acid Heme 23
Lipids 17
TCA/ Oxidative 19
Amino acid 20
Glucose/ Glycolysis 22
Total = 101
4 sgRNAs per targets
8 non-target control sgRNAs
4 positive control sgRNAs (Tsc2)
B 4T1 EMT6 Control Arg2KO Control Arg2KO
150 – CAS9
37 – ARG2
50 – β Tubulin
C Liver X 4T1 EMT6 Control Arg2KO Control Arg2KO
150 – CAS9
50 – β Tubulin
37 – ARG1
(Long Exposure)
D Orthotopic injection of 4T1-Control vs 4T1-Arg2KO
BALB/c-Rag1–/–
14 days
Tumor resection
16 days
Harvest metastatic lungs
E Resected Tumors
Primary tumor weight (g)
ns
4T1-Control 4T1-Arg2KO
F Control Arg2KO
G Metastatic Lung Weight
Whole lung weight (g)
Lung Surface Tumor Nodules
Surface tumor nodule counts/mouse
H 4T1 Control Arg2KO;Empty Arg2KO;Arg2WT
37 – ARG2
1.00 0.04 0.96
50 – β Tubulin
I EO771.LMB Empty Arg2WT Arg2H160F
37 – ARG2
0.06 1.00 0.82
50 – β Tubulin
J Exon 1 Exon 4 Exon 8
G N I H G Q P
Arg2WT : GGAAATATACATGGACAGCCA
Arg2H160F : GGAAATATATTTGGACAGCCA
G N I F G Q P
160
K Arginase activity
Arginase activity (µmole/L urea/min/mg protein)
E0771.LMB-Empty E0771.LMB-Arg2WT E0771.LMB-Arg2H160F
L Orthotopic injection of EO771.LMB -Arg2WT vs -Arg2H160F
C57BL/6J
14 days
Tumor resection
5 weeks
Harvest metastatic lungs
M Resected Tumors
Primary tumor weight (g)
E0771.LMB-Arg2WT E0771.LMB-Arg2H160F
N EO771.LMB-Arg2WT EO771.LMB-Arg2H160F
O Metastatic Lung Weight
Whole Lung Weight (g)
Lung Tumor Burden
Percentage tumor lesion area
P Tail vein injection of 4T1-Control vs 4T1-Arg2KO
BALB/cF1-Cas9
3 weeks
Harvest metastatic lungs
Q Control Arg2KO
R Metastatic Lung Weight
Whole lung weight (g)
Lung Surface Tumor Nodules
Surface tumor nodule counts/mouse
4T1-Control 4T1-Arg2KO

Supplementary Figure 1. Validation of ARG2 expression and function in lung metastasis. A, The sgRNA library targets metabolic enzymes involved in glucose, nucleotide, heme, amino acid, and lipid metabolism, as well as the tricarboxylic acid (TCA) cycle and oxidative phosphorylation pathways. **B and C,** Western blot analysis of ARG2 (**B**) and ARG1 (**C**) protein expression in 4T1 and EMT6 cells with or without ARG2 deletion. Liver lysate serves as a positive control for ARG1 expression. **D-G,** Validation of ARG2 in regulating spontaneous lung metastasis in *Rag1*$^{-/-}$ mice. **D,** Experimental schematic for the model. **E,** Weights of resected 4T1 primary tumors (n = 12 per group). **F,** Representative gross lung images and H&E-stained lung sections showing metastatic burden. **G,** Whole lung weight and surface tumor nodule counts of harvested lungs. **H and I,** Western blot analysis of ARG2 expression in 4T1 (**H**) and EO771.LMB (**I**) cell lines following Arg2 knockout and re-expression, with quantification of relative intensity by densitometry. **J,** Schematic showing the H160F mutation in ARG2 and the corresponding nucleotide substitution. **K,** Arginase activity was determined in EO771.LMB cells expressing ARG2^{WT} or ARG2^{H160F}. **L-O,** *In vivo* spontaneous lung metastasis of EO771.LMB cells expressing ARG2^{WT} or ARG2^{H160F} in C57BL/6J mice. **L,** Experimental schematic of model. **M,** Weights of resected EO771.LMB primary tumors (n = 7 to 9 per group). **N,** Representative H&E-stained lung sections showing metastatic burden. **O,** Whole lung weight and surface tumor nodule counts of harvested lungs. **P-R,** Metastatic lung colonization and outgrowth following tail vein injection of 4T1-Control or 4T1-*Arg2*^{KO} cells. **P,** Experimental schematic of model. **Q,** Representative gross lung images and H&E-stained lung sections showing metastatic burden. **R,** Whole lung weight and surface tumor nodule counts of harvested lungs (n = 16 to 17 per group). Data are presented as mean ± SEM and analyzed using Welch's t-test or one-way ANOVA followed by Tukey's post hoc test. *, $p < 0.05$; **, $p < 0.01$. Scale bar is 200 µm for gross lung image and 1 mm H&E-stained lung sections.

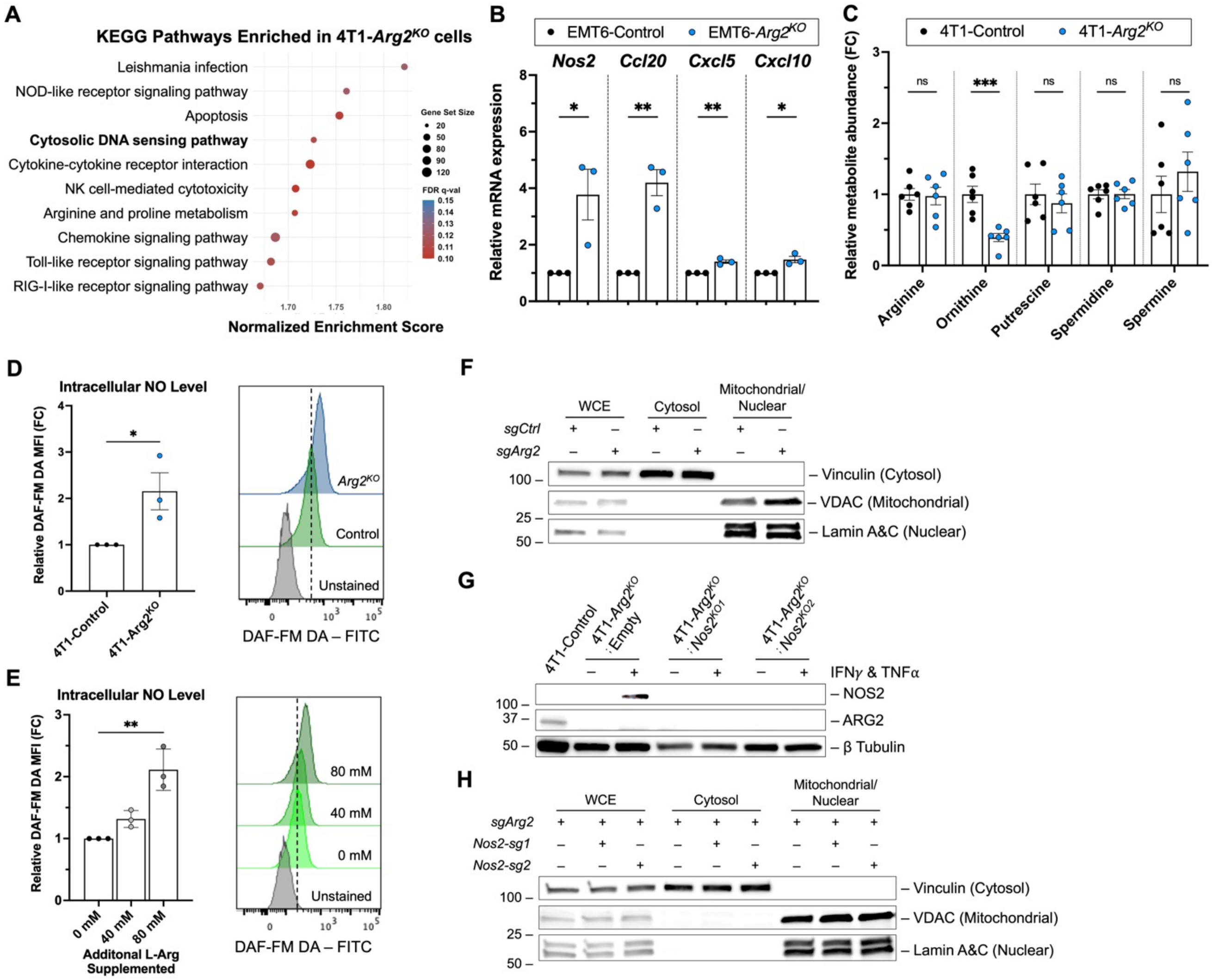

Supplementary Figure 2. Inflammatory signaling, metabolic alterations, and NOS2-dependent nitric oxide production in ARG2-deficient cells. A, KEGG pathway enrichment analysis of differentially expressed genes in 4T1-*Arg2*^{KO} cells compared with 4T1-Control cells. Gene set size and false discovery rate (FDR) *q*-value are indicated. **B** qRT-PCR analysis of NF-κB target genes (*Nos2, Ccl20, Cxcl5,* and *Cxcl10*) in EMT6-Control and EMT6- *Arg2*^{KO} cells (n=3 per group). **C,** Metabolomic analysis of arginine metabolism-related metabolites in 4T1-Control and 4T1-*Arg2*^{KO} cells (n=6 per group). **D and E,** Flow cytometry analysis of DAF-FM DA fluorescence to examine intracellular NO level in 4T1-Control and 4T1-*Arg2*^{KO} cells (**D**) or 4T1 cells cultured with or without additional L-arginine supplementation (**E**) (n=3 per group). **F,** Western blot analysis of subcellular fractions from control (sgCtrl) or *Arg2*^{KO} (sgArg2) 4T1 cells. Vinculin marks the cytosolic fraction, whereas VDAC and lamin A/C mark the mitochondrial and nuclear fractions, respectively. **G,** Western blot analysis confirming deletion of NOS2 in 4T1-*Arg2*^{KO}*;Nos2*^{KO} cells. Treatment with TNFα and IFNγ (20 ng/mL each) was used to induce maximal NOS2 expression. **H,** Western blot analysis of subcellular fractions from *Arg2*^{KO} (sgArg2) and *Arg2*^{KO}*;Nos2*^{KO} 4T1 cells, with fraction markers indicated. Two independent sgNos2 sequences were evaluated. Data are presented as mean ± SEM and analyzed using Welch's t-test *, $p < 0.05$; **, $p < 0.01$; ***, $p < 0.001$.

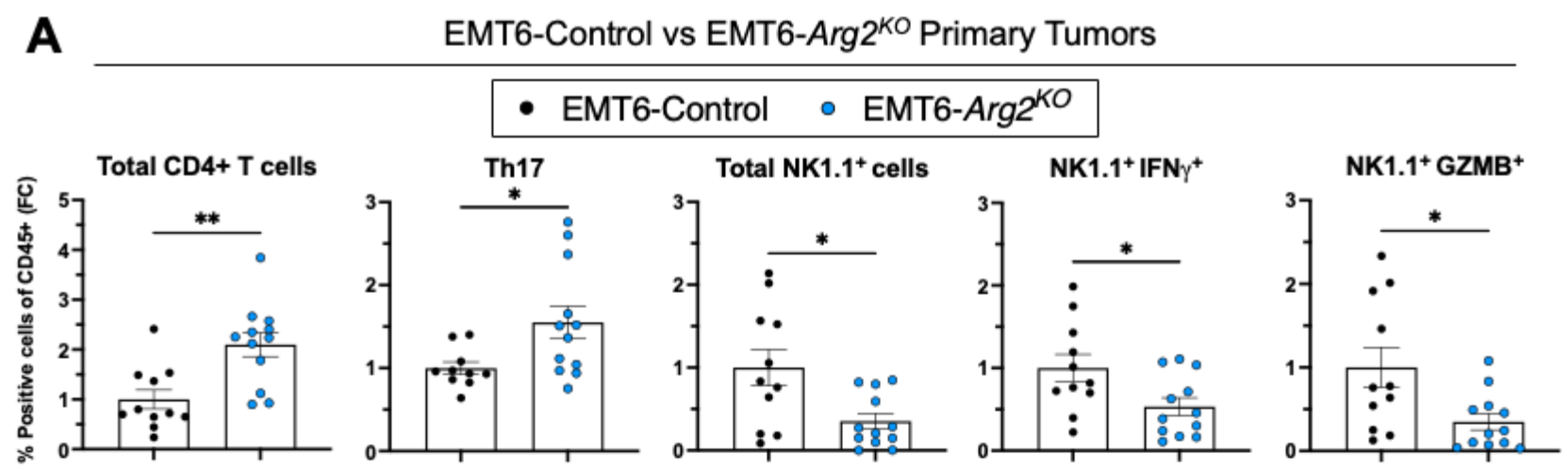

B

Upregulated EMT-associated genes in 4T1-*Arg2KO* cells (Hallmark – EMT)					
**Rank**	**Gene**	**log2 fold change**	***p* value**	**Rank Metric Score**	**Running ES**
1	*Cxcl5*	2.64	7.1E-10	3.144	0.0697
34	*Il6*	2.83	1.0E-03	1.918	0.1099
43	*Tgm2*	0.96	4.9E-06	1.729	0.1477
125	*Mmp3*	0.76	4.9E-05	1.162	0.1673
147	*Ccn1*	0.76	1.5E-07	1.077	0.1896
370	*Itgb5*	0.45	1.7E-03	0.698	0.2668
473	*Vegfc*	0.95	5.3E-07	0.616	0.2726
514	*Ccn2*	0.29	3.0E-02	0.591	0.2827
574	*Itga2*	0.64	1.1E-04	0.544	0.3154
764	*Tgfb1*	0.53	1.4E-04	1.918	0.3561
1112	*Serpine2*	1.06	6.0E-06	0.353	0.3820
1160	*Tgfbr3*	1.08	1.2E-05	0.339	0.3937
1206	*Inhba*	0.49	7.3E-04	0.327	0.3975

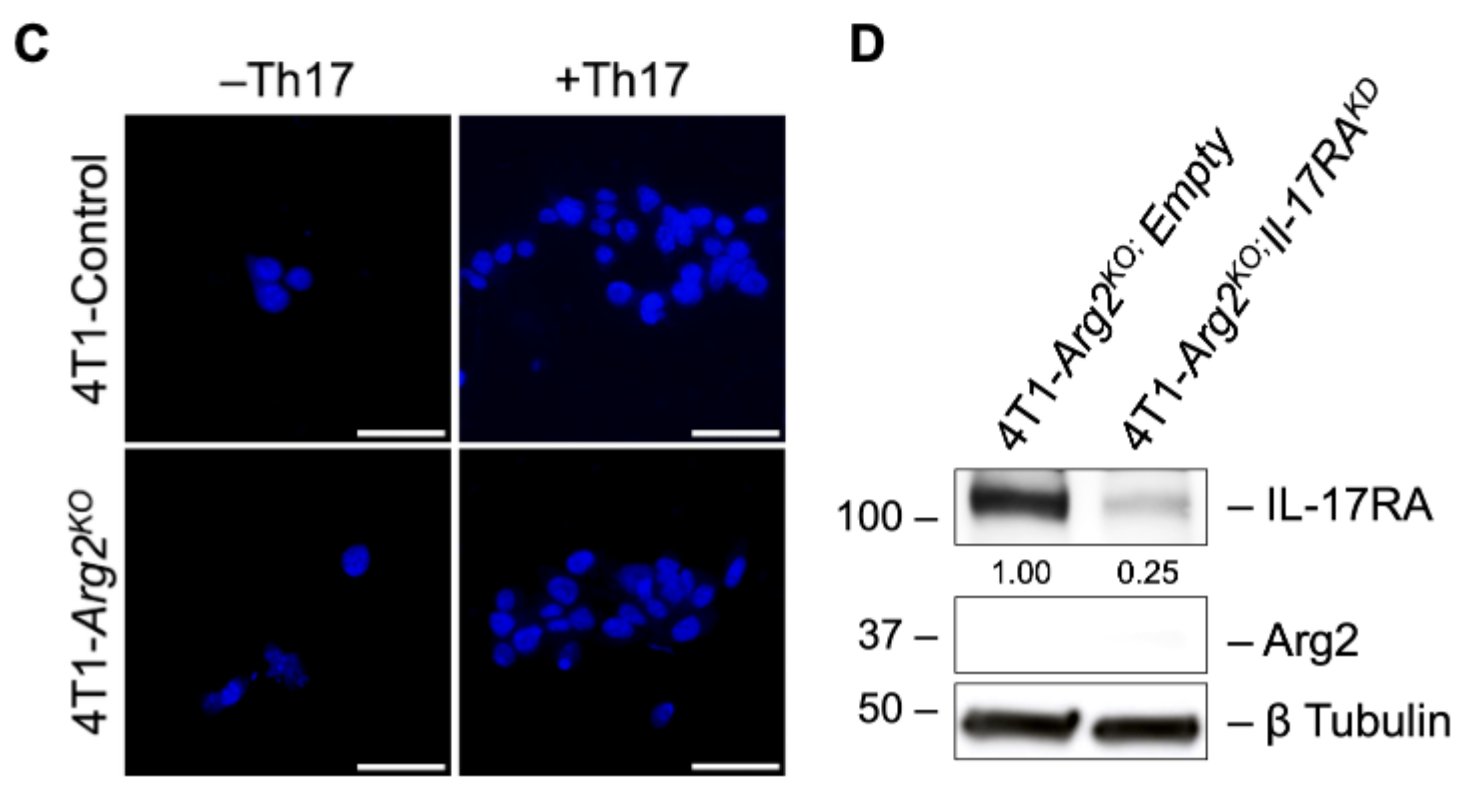

Supplementary Figure 3. Immune profiling of EMT6 tumors, 4T1 EMT-associated genes, and tumor cell migration in the presence of Th17 cells. A, Flow cytometric analysis of immune cell populations in EMT6-Control and EMT6-*Arg2*^{KO} primary tumors. Total CD4^{+} T cells, CD4^{+} IL-17A^{+} (Th17) cells, total NK1.1^{+} cells, NK1.1^{+}IFNγ^{+} cells, and NK1.1^{+}GZMB^{+} cells were determined as a percent (%) of CD45^{+} immune cells. Data are presented as fold change (FC) relative to the control (n=11 to 12 per group). **B,** Ranked list of upregulated EMT-associated genes identified by mRNA sequencing in 4T1-*Arg2*^{KO} cells from the EMT gene hallmark. Log$_2$ fold change, adjusted *p* value, rank metric score, and running enrichment score are indicated. **C,** Representative images of transwell migration assay of 4T1-Control or 4T1-*Arg2*^{KO} cells co-cultured with or without *in vitro*-differentiated Th17 cells. Nuclei of migrated cells were stained with DAPI. Scale bars are 50 µm. **D,** Western blot analysis confirming IL-17RA knockdown in 4T1-*Arg2*^{KO} cells. Relative densitometry values are shown. Data are presented as mean ± SEM and analyzed using Welch's t-test. *, $p < 0.05$; **, $p < 0.01$.

**Supplementary Table 1. Oligonucleotides, DNA fragments, and plasmids**		
**Oligonucleotides**		
**Gene Target**	**Sequence–Forward (5’→3’)**	**Sequence–Reverse (5’→3’)**
**Quantitative PCR Primers**		
Mouse Arg2	GGCTGATTGGCAAAAGGCAG	ATCCCCCTACAACAGGGGTT
Mouse Rpl37	CTACCGCAGATTCAGACATGGA	ACCGAACTCTGAACCGATGT
Mouse Nos2	AACTTTACAGGGAGTTGAAGACTGA	CAAATCCAACGTTCTCCGTTCT
Mouse Ccl20	GCAACTACGACTGTTGCCTC	ACATCTTCTTGACTCTTAGGCTGA
Mouse Il-6	TACCACTTCACAAGTCGGAGGC	CTGCAAGTGCATCATCGTTGTTC
Mouse Cxcl5	CCCTACGGTGGAAGTCATAGC	TTAGCTTTCTTTTTGTCACTGCCC
Mouse Cxcl10	GAAATCATCCCTGCGAGCCT	AGTTAAGGAGCCCTTTTAGACCTTT
Mouse Mmp3	ACATGGAGACTTTGTCCCTTTTG	TTGGCTGAGTGGTAGAGTCCC
Mouse Mmp13	GATGACCTGTCTGAGGAAGACC	GCATTTCTCGGAGCCTGTCAAC
Mouse mtD-loop1	AATCTACCATCCTCCGTGAAACC	TCAGTTTAGCTACCCCCAAGTTTAA
Mouse mtD-loop2	CCCTTCCCCATTTGGTCT	TGGTTTCACGGAGGATGG
Mouse mt-16S	CACTGCCTGCCCAGTGA	ATACCGCGGCCGTTAAA
Mouse mt-Nd4	AACGGATCCACAGCCGTA	AGTCCTCGGGCCATGATT
**CRISPR sgRNA**		
Arg2-sgRNA	caccgCCAAATATTGTGTACATTGG	aaacCCAATGTACACAATATTTGGc
LacZ-sgRNA	caccgTGCGAATACGCCCACGCGAT	aaacATCGCGTGGGCGTATTCGCAc
Nos2-sgRNA1	caccgGTGACGGCAAACATGACTTC	aaacGAAGTCATGTTTGCCGTCACc
Nos2-sgRNA2	caccgGAGTTCATCAACCAGTATTA	aaacTAATACTGGTTGATGAACTCc
**Lentiviral Vectors and Transfection/Transduction Reagents**		
**Vector**	**Source**	**Identifier**
plentiCRISPRv2-Puro	Addgene	#52961
plentiCRISPRv2-Blast	Addgene	#98293
plenti-CMV-Blast	Addgene	#17486
pCMV-VSV-G	Addgene	#8454
psPAX2	Addgene	#12260
Lipofectamin™ 3000 Transfection Reagent	Thermo Fisher Scientific	#L3000-015
Polybrene	Sigma-Aldrich	#TR-1003-G
**gBlock DNA**		
**Gene Target**	**Sequence (5’→3’)**	
Arg2[WT]	cctccatagaagacaccgacTCTAGAGCCACCATGTTCCTGAGGAGCAGCGCCTCCCGTCTCC<br>TCCACGGGCAAATTCCTTGCGTCCTGACGAGATCCGTCCACTCTGTAGCTATAGTC<br>GGAGCCCCTTTCTCTCGGGGACAGAAGAAGCTAGGAGTGGAATATGGTCCAGCTG<br>CCATTCGAGAAGCTGGCTTGCTGAAGAGGCTCTCCAGGTTGGGATGCCACCTAAA<br>AGACTTTGGAGACTTGAGTTTTACTAATGTCCCACAAGATGATCCCTACAATAATC<br>TGGTTGTGTATCCTCGTTCAGTGGGCCTTGCCAACCAGGAACTGGCTGAAGTGGTT<br>AGTAGAGCTGTGTCAGGTGGCTACAGCTGTGTCACCATGGGAGGAGACCACAGCC<br>TGGCAATAGGTACCATTATCGGTCACGCCCGGCACCGCCCAGATCTCTGTGTCATC<br>TGGGTTGATGCTCATGCGGACATTAATACACCTCTCACCACTGTATCTGGAAATAT<br>ACATGGACAGCCACTTTCCTTTCTCATCAAAGAACTACAAGACAAGGTACCACAA<br>CTGCCAGGATTTTCCTGGATCAAACCTTGCCTCTCTCCgCCcAATATaGTcTACATTG<br>GCCTGAGAGATGTGGAGCCTCCTGAACATTTTATTTTAAAGAATTATGACATCCA<br>GTATTTTTCCATGAGAGAGATTGATCGACTTGGGATCCAGAAGGTGATGGAACAG<br>ACATTTGATCGGCTGATTGGCAAAAGGCAGAGGCCAATCCACCTGAGTTTTGACA<br>TTGATGCATTTGACCCTAAACTGGCTCCAGCCACAGGAACCCCTGTTGTAGGGGG<br>ATTAACCTACAGAGAAGGAGTGTATATTACTGAAGAAATACATAATACAGGGTTG<br>CTGTCAGCTCTGGATCTTGTTGAAGTCAATCCTCATTTGGCCACTTCTGAGGAAGA<br>GGCCAAGGCAACAGCCAGACTAGCAGTGGATGTGATTGCTTCAAGTTTTGGTCAG<br>ACAAGAGAAGGAGGACACATTGTCTATGACCACCTTCCTACTCCTAGTTCACCAC<br>ACGAATCAGAAAATGAAGAATGTGTGAGAATTTAGgtcgacaatcaacctctggattacaa	

Arg2^{H160F}	cctccatagaagacaccgacTCTAGAGCCACCATGTTCCTGAGGAGCAGCGCCTCCCGTCTCC<br>TCCACGGGCAAATTCCTTGCGTCCTGACGAGATCCGTCCACTCTGTAGCTATAGTC<br>GGAGCCCCTTTCTCTCGGGGACAGAAGAAGCTAGGAGTGGAATATGGTCCAGCTG<br>CCATTCGAGAAGCTGGCTTGCTGAAGAGGCTCTCCAGGTTGGGATGCCACCTAAA<br>AGACTTTGGAGACTTGAGTTTTACTAATGTCCCACAAGATGATCCCTACAATAATC<br>TGGTTGTGTATCCTCGTTCAGTGGGCCTTGCCAACCAGGAACTGGCTGAAGTGGTT<br>AGTAGAGCTGTGTCAGGTGGCTACAGCTGTGTCACCATGGGAGGAGACCACAGCC<br>TGGCAATAGGTACCATTATCGGTCACGCCCGGCACCGCCCAGATCTCTGTGTCATC<br>TGGGTTGATGCTCATGCGGACATTAATACACCTCTCACCACTGTATCTGGAAATAT<br>AtttGGACAGCCACTTTCCTTTCTCATCAAAGAACTACAAGACAAGGTACCACAACT<br>GCCAGGATTTTCCTGGATCAAACCTTGCCTCTCTCCgCCcAATATaGTcTACATTGG<br>CCTGAGAGATGTGGAGCCTCCTGAACATTTTATTTTAAAGAATTATGACATCCAGT<br>ATTTTTCCATGAGAGAGATTGATCGACTTGGGATCCAGAAGGTGATGGAACAGAC<br>ATTTGATCGGCTGATTGGCAAAAGGCAGAGGCCAATCCACCTGAGTTTTGACATT<br>GATGCATTTGACCCTAAACTGGCTCCAGCCACAGGAACCCCTGTTGTAGGGGGAT<br>TAACCTACAGAGAAGGAGTGTATATTACTGAAGAAATACATAATACAGGGTTGCT<br>GTCAGCTCTGGATCTTGTTGAAGTCAATCCTCATTTGGCCACTTCTGAGGAAGAGG<br>CCAAGGCAACAGCCAGACTAGCAGTGGATGTGATTGCTTCAAGTTTTGGTCAGAC<br>AAGAGAAGGAGGACACATTGTCTATGACCACCTTCCTACTCCTAGTTCACCACAC<br>GAATCAGAAAATGAAGAATGTGTGAGAATTTAGgtcgacaatcaacctctggattacaa

**Supplementary Table 2. Western blot and flow cytometry antibodies**		
**Reagent or Resource**	**Source**	**Identifier**
**Western Blot Antibodies**		
ARG2	Abcam	#ab228963
ARG1	Cell Signaling Technology	#93668
β-tubulin	Sigma-Aldrich	#T4026
Cas9	Cell Signaling Technology	#14697
NOS2 (iNOS)	Cell Signaling Technology	#13120
NF-κB-p65	Cell Signaling Technology	#8242
Phospho-NF-κB-p65 (S536)	Cell Signaling Technology	#3033
STING	Cell Signaling Technology	#50494
Phospho-STING (S365)	Cell Signaling Technology	#9234
Vinculin	Cell Signaling Technology	#13901
VDAC	Cell Signaling Technology	#4661
Lamin A+C	Abcam	#ab169532
Anti-rabbit IgG HRP-linked	Cell Signaling Technology	#7074
Anti-mouse IgG HRP-linked	Cell Signaling Technology	#7076
**Flow Cytometry Antibodies and Viability Dye**		
CD45–APC-Cy7 (30-F11)	BD Biosciences	#557659
TCRβ–PE-Cy7 (H57-597)	Cytek Biosciences	#60-5961
CD8–redFluor 710 (53-6.7)	Cytek Biosciences	#80-0081
CD4–PE/Dazzle 594 (GK1.5)	BioLegend	#100455
NK1.1–Brilliant Violet 605 (PK136)	BioLegend	#108739
IL-17A–PerCP/Cyanine5.5 (TC11-18H10.1)	BioLegend	#506920
IFNγ–violetFluor 450 (XMG1.2)	Cytek Biosciences	#75-7311
Granzyme B–PE (NGZB)	Thermo Fisher Scientific	#12-8898-82
TNFα–APC (MP6-XT22)	Thermo Fisher Scientific	#17-7321-82
Ghost Dye Violet 510	Cytek Biosciences	#13-0870
Anti-CD16/32 Mouse Fc Block	Cytek Biosciences	#70-0161
